# The Anti-Tumor Agent Sodium Selenate Decreases Methylated PP2A, Increases GSK3βY216 Phosphorylation, Including Tau Disease Epitopes and Reduces Neuronal Excitability in SHSY-5Y Neurons

**DOI:** 10.3390/ijms20040844

**Published:** 2019-02-15

**Authors:** Wesal Habbab, Imad Aoudé, Freshteh Palangi, Sara Abdulla, Tariq Ahmed

**Affiliations:** The Neurological Disorders Research Center, Qatar Biomedical Research Institute, Hamad Bin Khalifa University, P.O. Box: 5825 Al Rayyan, Ad Dawḩah, Doha, Qatar; whabbab@hbku.edu.qa (W.H.); iaoude@hbku.edu.qa (I.A.); fpalangi@hbku.edu.qa (F.P.); saabdulla@hbku.edu.qa (S.A.)

**Keywords:** PP2A, GSK3β, Tau, retinoic acid differentiated SHSY-5Y cells

## Abstract

Selenium application as sodium selenate was repeatedly shown to have anti-carcinogenic properties by increasing levels of the serine/ threonine protein phosphatase 2A (PP2A) in cancer cells. PP2A has a prominent role in cell development, homeostasis, and in neurons regulates excitability. PP2A, GSK3β and Tau reside together in a complex, which facilitates their interaction and (dys)-function as has been reported for several neurological disorders. In this study we recorded maximum increase in total PP2A at 3 µM sodium selenate in a neuron cell line. In conjunction with these data, whole-cell electrophysiological studies revealed that this concentration had maximum effect on membrane potentials, conductance and currents. Somewhat surprisingly, the catalytically active form, methylated PP2A (mePP2A) was significantly decreased. In close correlation to these data, the phosphorylation state of two substrate proteins, sensitive to PP2A activity, GSK3β and Tau were found to be increased. In summary, our data reveal that sodium selenate enhances PP2A levels, but reduces catalytic activity of PP2A in a dose dependent manner, which fails to reduce Tau and GSK3β phosphorylation under physiological conditions, indicating an alternative route in the rescue of cell pathology in neurological disorders.

## 1. Introduction

Selenium is a non-metallic trace element which is incorporated into cells as a component of selenoproteins [1], including the anti-oxidant enzymes: glutathione peroxidases (GP) and thioredoxin reductases (TR), and the metabolic enzyme S-adenosylhomocysteine (S-ACH) [1,2,3,4]. Sodium selenate or other selenium derivatives such as selenomethionine, treatment was found to increase expression of the serine/threonine protein phosphatase 2A (PP2A) in cells and mitigate Tau pathology in rodent models of tauopathies, traumatic brain injury (TBI), Alzheimer’s disease (AD), in addition to rescuing behavioral phenotypes and synaptic plasticity in these models [5,6,7,8,9,10,11,12,13]. The PP2A—holoenzyme is the major protein phosphatase in neuronal cells and accounts for approximately 70% of the total phosphatase activity [14,15,16]. Under physiological condition, PP2A regulates a plethora of signalling cascades including the PI3K/AKT/GSK3β and Ras-MAPK pathways [17,18,19,20]. In AD [21]; Parkinson’s disease (PD) [22]; and Huntingdon’s disease (HD) [23]; over-activity of these serine/ threonine kinases or dysfunction in PP2A activity is linked with pathophysiology. Genetic ablation of PP2A is embryonic lethal in mice; and in human subjects PP2A dysfunction can result in learning problems, intellectual disability [24,25,26], and is closely associated with loss of cognitive flexibility observed in AD subjects [15,27,28].

The PP2A holoenzyme is composed of a 36 kDa catalytic subunit (PP2Ac) and regulatory and binding subunits PR55B (52 kDa) and PR61B ((either α, β, ε): 52, 52 and 55 kDa) respectively, these latter proteins affect cellular localization and binding to organelles in a site specific manner [16,20]. Intriguingly the microtubule protein Tau was found to be a cellular scaffold for PP2A—with implications for tauopathies and AD [29,30,31]. PP2A itself is tightly regulated by phosphorylation and methylation [16,18,32,33]. Phosphorylation by multiple kinases (e.g., PKA, MAPK) at PP2Ac-Tyr307 inhibits the recruitment of the PR55B and PR61B (including the αβε isotypes) family members to the core enzyme and its association with Tau [16,24,30,34,35]. Similarly, the C-terminal tail can also be phosphorylated at Thr304, which was found to inhibit the binding of PR55B subunits, but not the other B subunits [36]. PP2Ac is also subject to post-translation regulation by methylation at Leu309 (mePP2A), by Leucine carboxyl methyltransferase 1 (LCMT1). This enzyme was required only for the binding of certain regulatory B subunits and results in enhanced catalytic activity. The methylation of the C-terminal tail can be reversed by the specific phosphatase methylesterase 1(PME1) [16,37,38]. Unsurprisingly, dysregulation of these latter two enzymes was found in AD and Tauopathies; further indicating a pivotal role for PP2A in neuropathologies [3,18,28]. Moreover, and somewhat unsurprising was that in different types of cancers, PP2A dysfunction was also a common feature [19,39,40,41], and that sodium selenate has been routinely used as a tumor suppressor [42,43,44,45,46,47,48,49].

The optimum concentration of sodium selenate (Na_2_SeO_4_) that mediates enhanced PP2A activity in neurons remains unclear [12]. In an attempt to address this and other questions, we explored the effects of different doses of sodium selenate on PP2A levels in a model cell system: retinoic acid differentiated SHSY-5Y neurons [50]. In summary, our data reveal that sodium selenate enhances PP2A levels, but reduces catalytic activity in a dose dependent manner (with a maximum effect at 3 µM), which fails to reduce Tau and GSK3β phosphorylation under physiological conditions, indicating an alternative route to rescue of AD pathology.

## 2. Results

### 2.1. PP2A Levels Alter in Cells After Sodium Selenate Treatment

**Figure 1:**
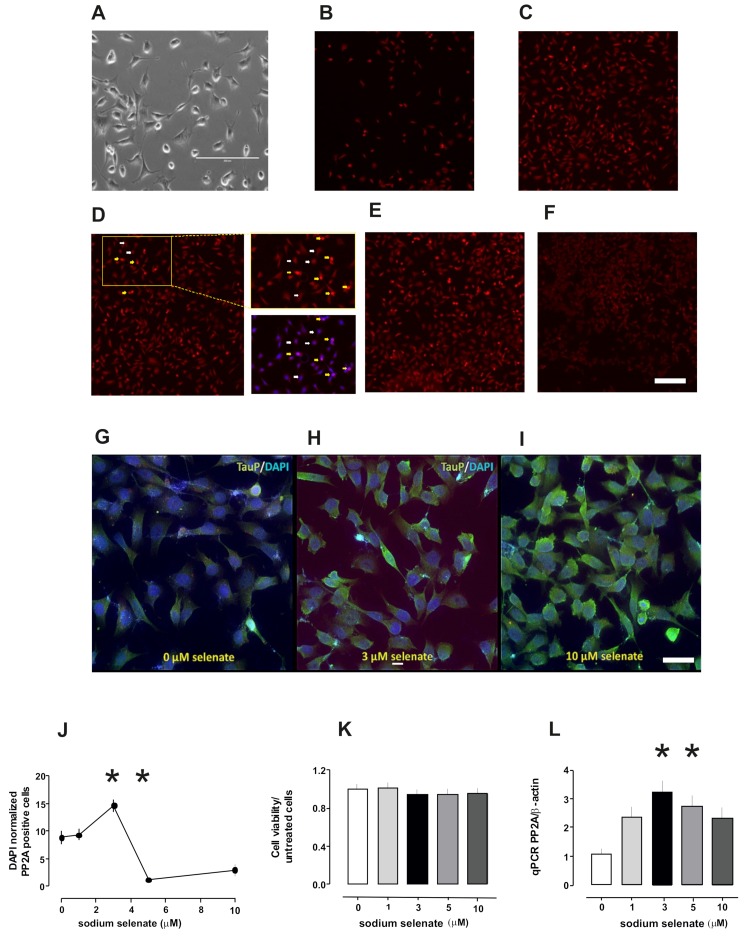
Sodium selenate treatment increases PP2Ac levels in differentiated SHSY-5Y cells without affecting cell viability. (**A**) A representative image of retinoic acid differentiated SHSY-5Y neurons with processes (scale bar 200 µm). (**B**) Sodium selenate treatment (0 µM) (**C**) 1 µM (**D**) 3 µM (**E**) 5 µM and (**F**) 10 µM, representative images of SHSY-5Y cells incubated with sodium selenate and stained with PP2Ac (red). Inset from (**D**) (yellow box) indicates two population of PP2A-positive cells with differential level of expression. High PP2A expression (yellow arrow) and low expression (white arrow). (**G**–**I**) Merged images of cells stained with nuclear marker DAPI (blue) and pTau (S202) (green) for 0, 3 and 10 µM sodium selenate treatment. Scale bar in top images (**F**) 100 µm; lower images (**I**) 50 µm). (**J**) Line-plot of PP2Ac positive cells for different doses of sodium selenate. **K.** Histogram plot of cell viability after different sodium selenate treatment. (**L**) Histogram plot qPCR of *PP2Ac* transcripts with sodium selenate treatment (asterisks *p* < 0.05, see also text).

Previous studies documented that 10 µM sodium selenate (Na_2_SeO_4_) was sufficient to enhance PP2A activity and dephosphorylate specific Tau epitopes in SHSY-5Y cells expressing human tau isoforms [5,12]. In a set of studies we found that 5 µM sodium selenate was sufficient to ameliorate synaptic plasticity in vitro in a Thy-Tau22 mouse line, which had reduced PP2A activity [13]. Furthermore, in in vivo studies 12 µg/L of sodium selenate in the drinking water was sufficient to correct some memory impairments in Tau and 3xTg AD mouse models [5,51]. Therefore, to gain a better understanding of the precise dose of sodium selenate that enhanced cellular PP2A activity, we monitored PP2Ac levels in retinoic acid differentiated SH-SY5Y cells after treatment with different doses of sodium selenate: 1, 3, 5 and 10 µM (Figure 1). The cytochemistry studies revealed enhanced PP2Ac positive cells, with an optimum treatment range of 3 µM selenate (*p* = 0.046), lower than previously reported [12,13] with a significant decrease in PP2Ac positive cells from this value at 5 µM (*p* = 0.002) and 10 µM sodium selenate (*p* = 0.0001; Figure 1). To establish that sodium selenate treatment was not effecting cell viability, we quantified viable and non-viable cells using flow-cytometery. In all conditions, we did not detect significant differences between the untreated cells (Figure 1). Next, we quantified expression levels of *PP2Ac* with RT-PCR experiments with *β-Actin* as controls for the different doses. Here we found *PP2Ac* peak expression starting at 3 µM that was twice untreated cells and was statistically significant (2.14 ± 0.6, *p* = 0.045; Figure 1) all normalized to *β-actin* levels. 

**Figure 2:**
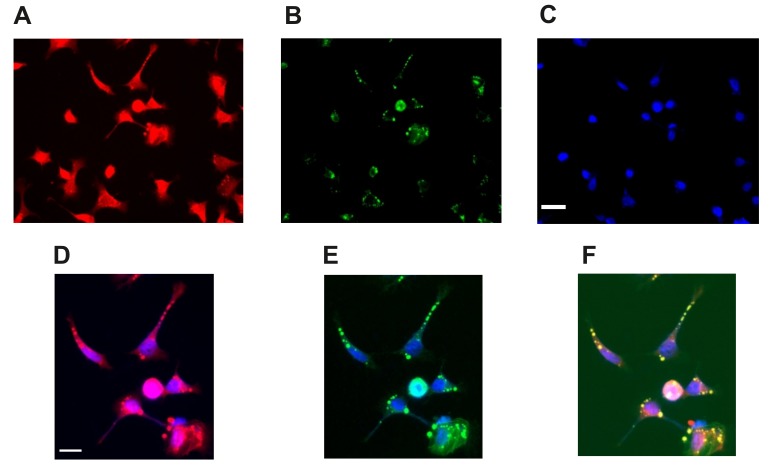
PP2Ac and pTau colocalize extranuclearin differentiated SHSY-5Y neurons. (**A**) Differentiated SHSY-5Y labelled with anti PP2A (red), (**B**) Same neurons labelled with anti-pTau (S202, green) and (**C**) labelled with nuclear stain DAPI (blue). (**D**) Merged image of PP2A and DAPI. (**E**) Merged image of pTau and DAPI and (**F**) Merged image of all three (yellow) highlighting co-localization of PP2A and pTau as extranuclear. Scale bar image (**C**) 50 µm and image (**D**) 20 µm.

To establish the cellular loci of PP2Ac and Tau we stained differentiated (untreated sodium selenate) SHSH-5Y cells with antibodies against these two proteins, counterstained with DAPI. Here we observed that PP2A and Tau co-localize outside the nucleus in these cells (Figure 2).

### 2.2. Active PP2A Decreases, Whilst Total GSK3β and Tau, Including Phosphorylation States Increase with Sodium Selenate Treatment

**Figure 3:**
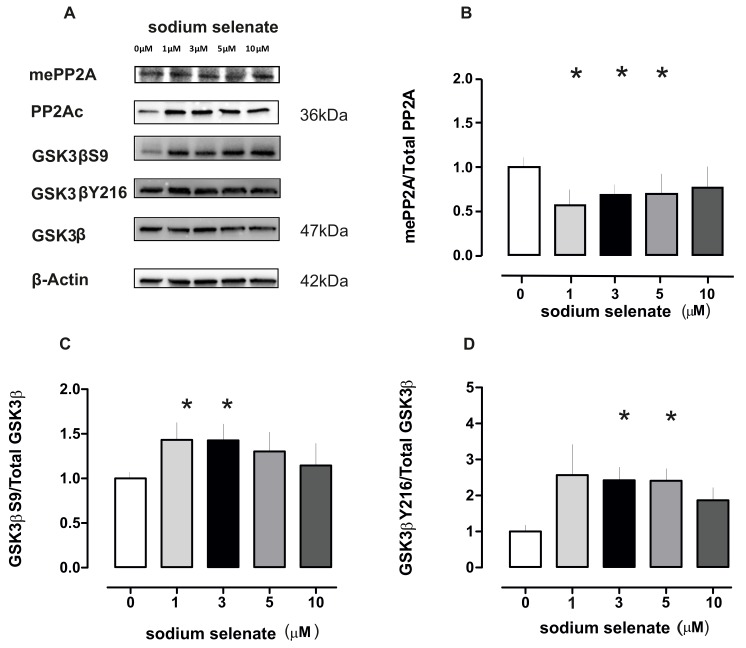
Sodium selenate treatment reduces active PP2Ac (mePP2A) and results in an increase in GSK3β phosphorylation at both inhibitory S9 and catalytic Y216 sites. (**A**) Immunoblot images of mePP2A, PP2Ac, (both 36 kDa) total GSK3β, GSK3βS9, GSK3βY216 (all 47 kDa) and loading control β-actin (42 kDa) for the different selenate dose. (**B**) Histogram plot of mePP2A-levels normalized to PP2Ac and β-actin values for different selenate doses. (**C**) Histogram plot of GSK3βS9/total GSK3β after different selenate doses. (**D**) Histogram plot of GSK3βY216/ total GSK3β after different selenate doses (in all plots asterisk *p* < 0.05).

In a series of immunoblots we confirmed that PP2Ac (36 kDa) increased in all treatments normalized to β-actin levels. Surprisingly when mePP2Ac was quantified and normalized to the PP2Ac/β-actin values we recorded a significant decrease at 1, 3 and 5 µM sodium selenate treatment (*p* = 0.016; *p* = 0.030; and *p* = 0.043 respectively; Figure 3 see also Supplementary Figure S1). 

One enzyme that is a substrate of and correlates well with active PP2A and, furthermore is involved in cellular homeostasis is GSK3β (47 kDa). Therefore, we investigated the phosphorylation states of GSK3β-ser9 (GSK3βS9), GSK3β-tyr216 (GSK3βY216), under sodium selenate treatment. For GSK3βS9 we recorded significant increase at 1 and 3 µM (*p* = 0.046 and *p* = 0.040 respectively). For GSK3βY216 we detected significant increases at 3 and 5µM sodium selenate levels (*p* = 0.009 and *p* = 0.007), all values were normalized to the total GSK3β and β-actin (Figure 3, see also Supplementary Figure S2).

**Figure 4:**
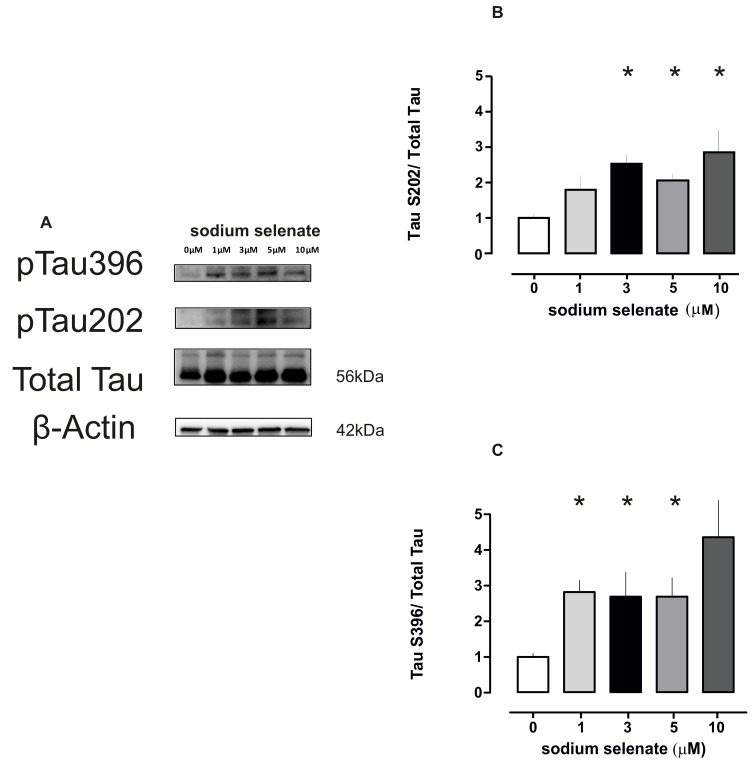
Sodium selenate treatment enhances total Tau and phosphorylation of two disease marker epitopes AT8 and PHF1 in a dose responsive manner. (**A**) Immunoblot images of Total Tau (56 kDa) and phosphorylated S202 (pTau202) and S396 (pTau396) epitopes for the different sodium selenate doses, loading control β-actin as above (not shown). (**B**,**C**) Histogram plots of pTauS202 and pTau396 after different selenate doses (asterisk *p* < 0.05).

The second substrate of PP2A and which is also a binding partner is Tau (56 kDa). For this molecule we investigated the phosphorylation states of Tau-ser202 and Tau-ser396 (the latter two are recognized as disease markers AT8-S202 and PHF1-S396) [52]. For the AT8 epitope we detected significant changes for 3, 5 and 10 µM sodium selenate (*p* = 0.009, *p* = 0.042 and *p* = 0.040, respectively). For the PHF-1 epitope significant changes were detected for 1, 3 and 5 µM (*p* = 0.003, *p* = 0.034 and *p* = 0.016 respectively, Figure 4, see also Supplementary Figure S3).

### 2.3. Electrophysiology Studies

**Figure 5:**
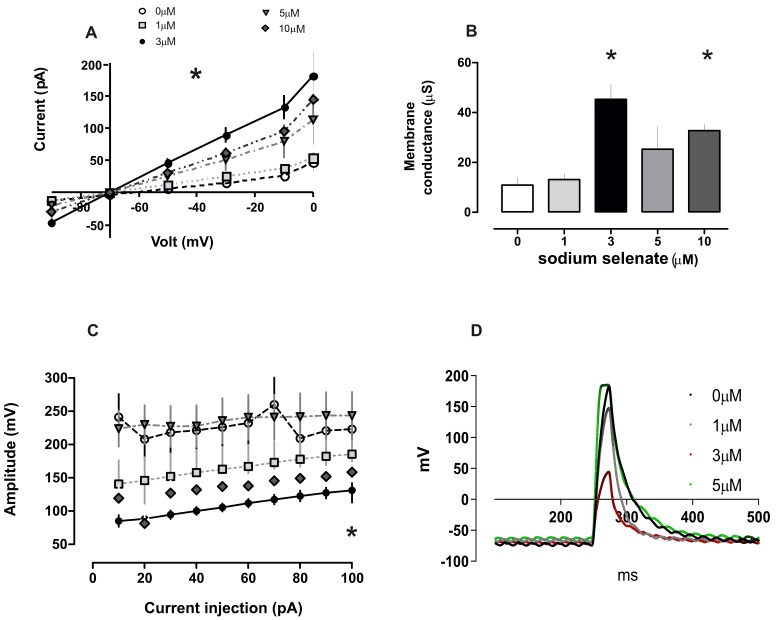
Sodium selenate alters membrane potential and conductance in a dose dependent manner**.** (**A**) Current-voltage (I-V) relationship for the effects of selenate dose on retinoic acid (RA) differentiated SHSY-5Y cells. (**B**) Bar-chart of membrane conductance after different selenate dose. (**C**) Membrane potentials after current injection in SHSY-5Y cells–indicative that 3 µM sodium selenate significantly dampens cell excitability (labelling as in **A**). In all sub-figures, asterisk *p* < 0.05. (**D**) Action potential (AP) changes induced with 90 pA current injection in RA differentiated SHSY-5Y cells after treatment with different selenate concentrations. Note that 3 µM sodium selenate has the maximum effect on AP.

The role of sodium selenate on neuronal excitability has, to our knowledge not been explored at the functional level. To achieve this, we recorded in voltage clamp mode (V_h_ −70 mV) current injection and established an I-V ratio, that was greatly enhanced for cells treated with 3 µM sodium selenate (comparing 0 and 3 µM, F(1,9) = 25.58, *p* = 0.0007, RM-ANOVA, Figure 5). In direct comparison, whole cell mode at −40 and +80 mV step values, yielded current values of −93 and 181 pA respectively, when compared with untreated cells at the same voltage steps −23 and 47 pA (*p* = 0.001, Figure 5). When the cell conductivity from different doses was plotted—we recorded that 3 µM sodium selenate increased membrane conductance: 45.3 ± 2.5 µS, compared with untreated cells of 10.9 ± 3.1 µS (*p* = 0.00057, *n* = 6 cell/group, *t*-test). Measurement of the magnitude of the evoked action potential (eAP) when current was injected into cells, again revealed that 3 µM sodium selenate treatment was the optimum concentration in dampening cell excitability—as clearly observed for 2 arbitrarily selected current injection steps 10 and 90 pA. The value for 0 µM sodium selenate at 10 pA was 241 ± 35 mV and at 90 pA, 223 ± 8.2 mV. In comparison for 3 µM sodium selenate, 10 pA was 85 ± 8.9 and 90 pA was 127 ± 8.5 mV (Figure 5). This dampening effect was clearly visible for the rise time (ms) for current injection at 10 and 90 pA as follows: 0 µM = 6.93 ± 1.8 and 8.2 ± 1.6, respectively; and 3 µM = 13.67 ± 0.9 and 11.8 ± 0.3 respectively (6 cell/group, Figure 5).

## 3. Discussion

Selenium is a trace element necessary in low doses for cell metabolism. For example, selenium derivatives such as selenocysteine and selenomethionine are incorporated into the production of anti-oxidant proteins GP and TR, which modulate reactive oxide species in cells and have been reported to induce neurogenesis in an AD murine model [1,4,53,54]. Sodium selenite (Na_2_SeO_3_), an alternate derivative was found to be more cytotoxic as it interferes with redox reactions in mitochondria [47,48,49]. Other studies have found that sodium selenate has anti-carcinogenic properties by increasing levels of the serine/threonine phosphatase PP2A in cancer cells-to antagonize the PI3K/AKT pathway [5,42,44]. Furthermore, PP2A itself has a prominent role in cell development, homeostasis, and in neurons regulates excitability. by dephosphorylation of kinase activated ion channels, returning the channel to the physiological “resting-state” [20,55,56,57]. PP2A, GSK3β and Tau reside together in a complex (see also Figure 2), which facilitates their interaction and (dys)-function [58,59,60]. This has major implications for numerous neurological diseases; for example, overactive GSK3β and dysregulation of PP2A were found to be clearly associated with several neuropathologies [61] (see introduction). Thus, our strategy was to define an optimum dose of sodium selenate that increases PP2A levels for possible cell therapy, in functional neurons; and measure membrane/electrical properties in these cells, a task not reported on before. Earlier studies identified that sodium selenate was not toxic upto 100 µM and reduced Tau phosphorylation in vivo, in hippocampal slice cultures and in neuroblastoma cells, and or cells overexpressing human Tau [5,12]. Here, we report that sodium selenate treatment resulted in increased mRNA and cellular PP2A-levels, with an optimum at 3 µM, followed by significant protein decrease at 5 and 10 µM. Amongst the novel findings was that methylated PP2A (mePP2A), the catalytically active enzyme, was decreased at 3 µM sodium selenate concentration and phosphorylation of endogenous Tau and active GSK3β increased (discussed below). As stated above both PP2A and GSK3β can regulate ion channel activity, which may have implications for neuronal activity [62,63]. Thus, a second novel finding in our study was that 3 µM sodium selenate had the maximum effect on membrane potentials and excitability: clearly seen in the I-V curve and the membrane conductance values (Figure 5; other parameters measured yielded similar results data not shown).

Sontag et al. [3,64] reported that selenate has at least a dual function, activation of PP2A, and inhibition of GSK3β. However, as outlined above, a decrease in mePP2A, and increases in Tau phosphorylation (at the two disease marker epitopes AT8 and PF1) and GSK3β phosphorylation at both epitopes (S9 and Y216) were found. These results seem counterintuitive, based upon published studies [5,12] (see also introduction) where an increase in PP2A was linked with a decrease of Tau and of phosphorylation at AT8 and PHF1 epitopes and of GSK3βS9. Further, several recent studies with the 3xTg AD mice subjected to selenomethionine in the drinking water for 12 weeks, revealed significant increase in PP2A and decrease in pGSK3βS9 phosphorylation state [54,65,66,67]. However, these authors failed to document mePP2A and pGSK3βY216 levels, arguably the two most relevant markers for enzyme activity (see introduction). For the latter, evidence is provided by a recent study that found that GSK3β activity is independent of S9 phosphorylation in mouse brain [68]. The observation that mePP2A decreased with dose, in our study, would point to an alternative mode of action of sodium selenate in AD and Tauopathies. In two recent proteomic studies involving 3xTg AD mice treated with either sodium selenate or selenomethionine, the authors detected increases in oxidative stress proteins: Thioredoxin reductase [66] and Peroxiredoxin [10], amongst a plethora of proteins involved in neuronal physiology and mitochondrial activity. These and other anti-oxidant proteins are known to regulate cell viability in different cell types and affect activation of GSK3β [69] This raises several questions regarding the role of sodium selenate, amongst them are: (i) was the increased PP2A mediated by sodium selenate a side effect? Our qPCR and immunoblot data would argue against this assumption, but without further investigation, we cannot be wholly certain; (ii) It should be mentioned that the two recent proteomic analyses (outlined above) documented no alterations in PP2A, this absence of PP2A change may be due to insensitivities in these assays [10,66]; (iii) A study from Zheng et al. [54] revealed that selenomethionine results in increases in PI3K/AKT/GSK3β and neurogenesis in hippocampi of AD mice. The SHSY-5Y cells in our study were differentiated with retinoic acid for process formation [50] and, as described above PP2A/Tau/GSK3β form a complex, which would be sufficient to account for changes in these enzymes. However, in AD and Tauopathies stressors are assumed to result in Tau hyperphosporylation and GSK3β hyperactivity with decreased phosphatase activity [13]; but the SHSY-5Y cells were not exposed to any stressor, or Tau activator. Then, how do we interpret the increased Tau hyperphosphorylation and GSK3βY216? Here, we argue, that as the SHSY-5Y cells were viable after different doses of sodium selenate, this enhanced phosphorylation in substrate proteins is part of a normal physiological response that is required for differentiating neurons.

In conclusion, sodium selenate enhances PP2A levels but acts through an alternate pathway to affect membrane potentials and dephosphorylate Tau and GSK3β with implications for therapies in neurodegenerative disease models.

## 4. Material Methods

### 4.1. Cell Culture

Neuroblastoma SHSY-5Y cells (ATCC CRL-2266, a kind gift from Dr. El-Agnaf, QBRI) were prepared and differentiated as described by Encinas et al. [50,70]. In brief, cells were plated 10,000 cells/2 mL of media (Dulbecco’s modified Eagle’s medium (DMEM) containing heat-inactivated bovine fetal serum 12% (F12), 5 mL glutamax and penicillin (20 units/mL), streptomycin (20 mg/mL). All trans-retinoic acid (RA) was added to the media at a final concentration of 10 µM (50% ethanol *v*/*v*) all material from Thermo Fisher Scientific, (Waltham, MA, USA). Cells were maintained at 37 °C in a saturated humid atmosphere containing 95% air and 5% CO_2_. After 5 days in the presence of RA, cells were washed three times with DMEM and incubated 24 h with sodium selenate (Sigma-Aldrich, St. Louis, MO, USA), dissolved in media to the required concentration (0, 1, 3, 5 and 10 µM).

### 4.2. Immunofluorescence Microscopy

In a 24 well plate, cover slips were pretreated with laminin 10 µg/mL overnight and cells were plated as outlined above. After sodium selenate treatments, cells were rinsed three times with phoshphate buffered saline (PBS) pH 7.4, then fixed in 4% para-formaldehyde for 30 min at room temp (RT) and then subsequently washed three times with TBST (TBS with 0.2% Tween pH 7.4). Fixed cells were then permeabilized using PBST (PBS with 0.2% Triton) for 10 min, washed three times with TBST, and incubated in blocking buffer (5% normal horse serum in PBS) with continuous shaking for at least 1 h at RT. After blocking, cells were incubated, with continuous shaking over night at 4 °C, with the desired primary antibodies that have been diluted in the blocking buffer. Afterwards, cells were washed 3 times with TBST, stained with appropriate secondary antibodies (1:2000 dilution) for an hour rocking at RT, were washed three times with TBST. Finally, 0.5 µg/mL Hoechst 33258 dye (“DAPI” Molecular probes) were added to the cells for nuclear staining. After washing with PBS, cover slips were mounted in polyvinyl mounting medium and the cells were viewed and photographed with Axio Ziess fluorescence microscope using 20× and 40× objectives. The number of PP2A positive cells were counted using image J software and PP2A density was normalized to the number of DAPI positive cells. In total for each condition and each replicate a total of six images with an area of 1136.72 × 912.02 µm were counted.

### 4.3. Flow Cytometery

Sodium selenate treated and untreated SHSY-5Y cells were trypsinized and suspended in 100 μL staining solution (PBS with 2% FBS and 0.1% sodium azide). Suspended cells were treated with 7AAD (BD Biosciences, San Jose, CA, USA) viability dye and incubated for 20 min at 4 °C to discriminate between live and dead cells. Subsequently cells were washed and resuspended in 1 mL flow cytometry staining buffer (Fetal Bovine Serum with sodium azide). Data were acquired with a BD FACSCanto II flow cytometer using BD FACSDiva software (BD Bioscience) and analyzed on BD FACSuite software (BD Biosciences).

### 4.4. qRT-PCR

Total RNA was isolated from tissue samples (Norgen Biotek Corp., Thorold, ON, Canada) and processed for cDNA preparation according to manufacturers’ instructions (Quantitect RT-PCR Kit, Qiagen, Dusseldorf, Germany). qReal-Time PCR was achieved with SYBRgreen mastermix (Thermo Fisher Scientific) with the optimized annealing temperature 60 °C:

*PP2C-*forward-GCCTCTGCGAGAAGGCTAAA and reverse-GAATGGTGATGCGTTCACGG [71] and *β-actin-*forward-AGAGCTACGAGCTGCCTGAC and reverse-AGCACTGTGTTGGCGTACAG with the latter used as a control. In the study Δ*C*t was obtained by subtracting the *C*t values of *β-actin* from the *C*t values of *PP2C*, then normalized to the former and plotted as a graph.

### 4.5. Immunoblotting

Total protein from cells was extracted using RIPA Buffer Solution (Thermo Fisher Scientific 89900) with Proteinase (P8465-5ML) and Phosphatase Inhibitor Cocktail 3 (P0044-1ML, both from Sigma-Aldrich) added as per manufacturers’ instructions. This cocktail was added to cultured cells at 200 μL/well on ice. Cell lysates were collected in 1.5 mL Eppendorf tubes, centrifuged at 14,000× *g*. The resulting supernatant was transferred to clean 1.5 mL tubes, from which protein concentrations were quantified in triplicate with BCA colorimetric assay with a BSA standard (Thermo Fisher Scientific BCA assay kit). For immunoblotting. total protein (15 μg) was fractionated through an SDS-PAGE gel (stacking 4–12% resolving) and transferred onto a 0.45 polyvinylidene difluoride membrane (Trans Blot Turbo Biorad Inc., Hercules, CA, USA). After transfer, membranes were washed in Tris-buffered saline containing 0.1% (*v*/*v*) Tween-20, (TBST) pH 7.4, blocked with 5% (*w*/*v*) non-fat dry milk in incubated with the relevant primary antibody (1:1000 dilution unless stated), at 4 °C on a rocking platform, overnight. For protein band detection blots were washed 3 × 10 min with TBST pH 7.4 and incubated with horse radish peroxidase (HRP)-linked secondary antibodies (Ab; 1:100,000) for 1 h at room temperature on a rocking platform, the proteins were detected using enhanced chemiluminescence reagent (Pierce, MA, USA) and imaged on a ChemDoc system (Biorad). The band densities of the images obtained were then quantified using the basic Quantity One software (Biorad). In studies here β-Actin was used as the loading control. For re-probing, blots were stripped with Restore Western Blot buffer, as per manufacturers’ instructions (Thermo Fisher Scientific) and to confirm no residual activity, blots were probed with secondary—HRP-linked Ab alone and imaged to confirm complete removal of previously tested primary Ab.

### 4.6. Antibodies

We evaluated expression of catalytic subunit (total) PP2A (PP2Ac, 1/1000 in TBST) as well as methylated (Leu 309) PP2Ac (mePP2Ac, 1/1000; Biolegend, San Diego, CA, USA). mePP2Ac level was interpreted as an index of PP2Ac activity. The following antibodies GAPDH, β-Actin were purchased from Cell Signaling Technologies (CST, Danvers, MA, USA). The following antibodies were purchased from Abcam Ltd. (Cambridge, UK): total GSK3β, GSK3β39, GSK3βY216, Total Tau, Tau 231, Tau S396 (all 1/1000 in TBST).

### 4.7. Electrophysiology

The SHSY-5Y neurons were visualized through an inverted microscope (Olympus IX53) equipped with an Olympus DP27 camera and a 40× objective (numerical aperture, 0.55). Electrophysiological recordings were with an EPC-10 USB amplifier combined with Patchmaster software (HEKA Electronic, Ludwigshafen, Germany). Whole-cell patch clamp recordings were at room temperature (23 °C) on retinoic acid differentiated SHY5Y neurons. Recording electrodes were pulled from a P-97 pipette puller (Sutter Instrument Co., Novato, CA, USA), using borosilicate glass micropipettes (A-M: Systems, Siena, Italy) and had a resistance of 5–8 MΩ. Composition of solutions for the recording of membrane properties and evoked action potentials, were composed of (mM): 144 K^+^-gluconate, 10 KCl, 10 HEPES, and 2 Mg^2+^-ATP, and pH 7.20 adjusted with KOH. The extracellular recording solutions were composed of (mM): 140 NaCl, 5.5 KCl, 1 MgSO_3_·6H_2_O, 2 CaCl_2_·2H_2_O, 15 HEPES, 10 Glucose, and the pH 7.4. The junction potential was ~15 mV and corrected in all experiments. The membrane potentials, under voltage-clamp condition were held at −70 mV and currents were evoked by stepping the membrane potential in series from −40 to +80 mV (10 mV increments) for 50 ms. Action potentials, under current-clamp condition with membrane potential around −70 mV, were evoked by injecting a series of current steps from 10 to 100 pA (in 10 pA increments) for 50 ms, signals were sampled at 50 kHz and filtered at 10 kHz.

### 4.8. Statistics

Unless otherwise stated all studies were in triplicate with statistical analyses (GraphPad Inc., San Diego, CA, USA) *t*-tests with Welch’s correction, *p* values noted in the text.

## Supplementary Materials

Supplementary materials can be found at http://www.mdpi.com/1422-0067/20/4/844/s1.
