# Supplementary material for: The Anti-Tumor Agent Sodium Selenate Decreases Methylated PP2A, Increases GSK3βY216 Phosphorylation, Including Tau Disease Epitopes and Reduces Neuronal Excitability in SHSY-5Y Neurons
Source: Int J Mol Sci. 2019 Feb 15;20(4):844. doi: 10.3390/ijms20040844 (PMC6412488; doi:10.3390/ijms20040844)

Supplementary figure 1 PP2A alterations after selenate treatment

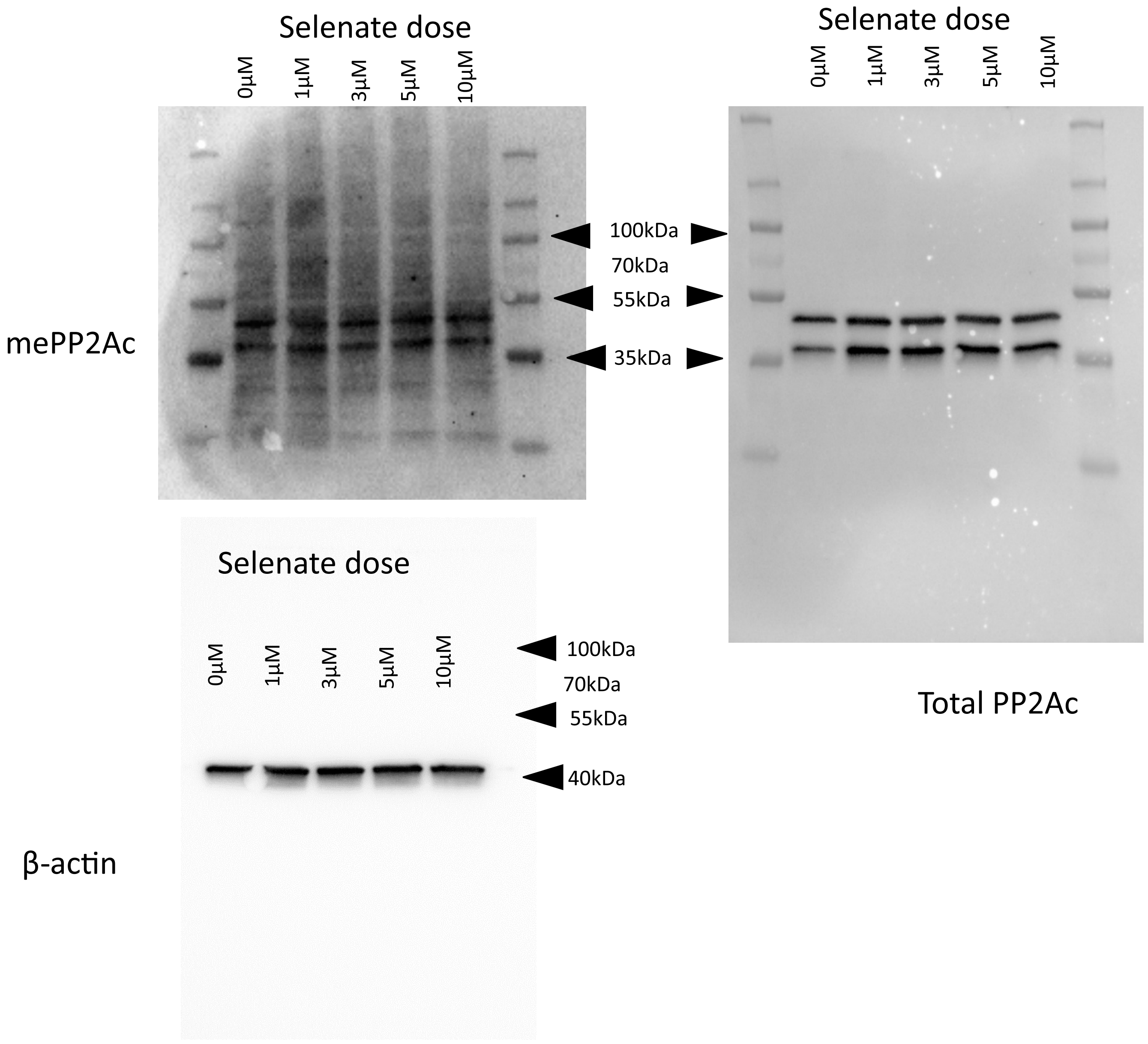

Supplementary figure 2 GSK3β changes after selenate treatment

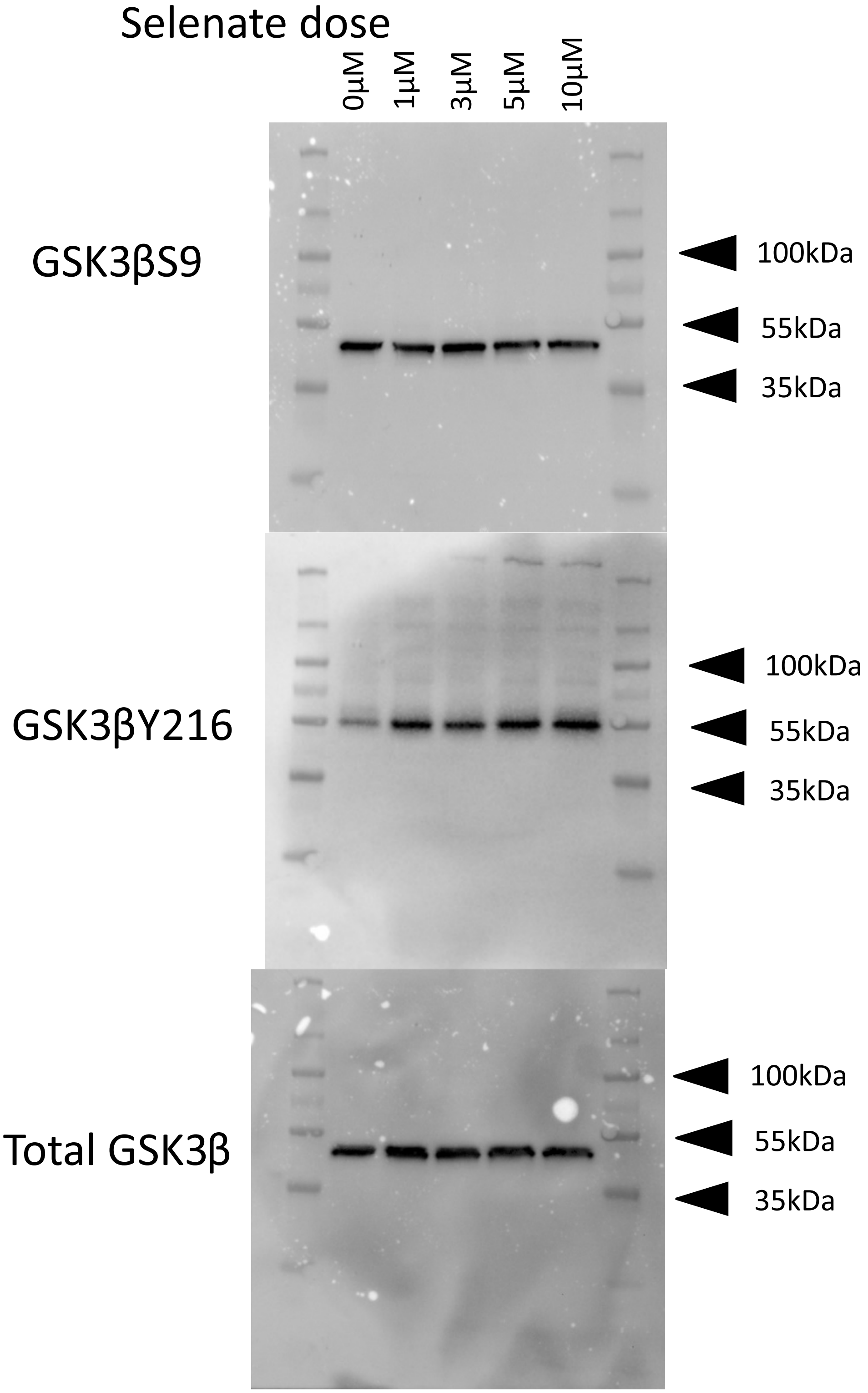

Supplementary figure 3 Tau phosphorylation changes after selenate treatment

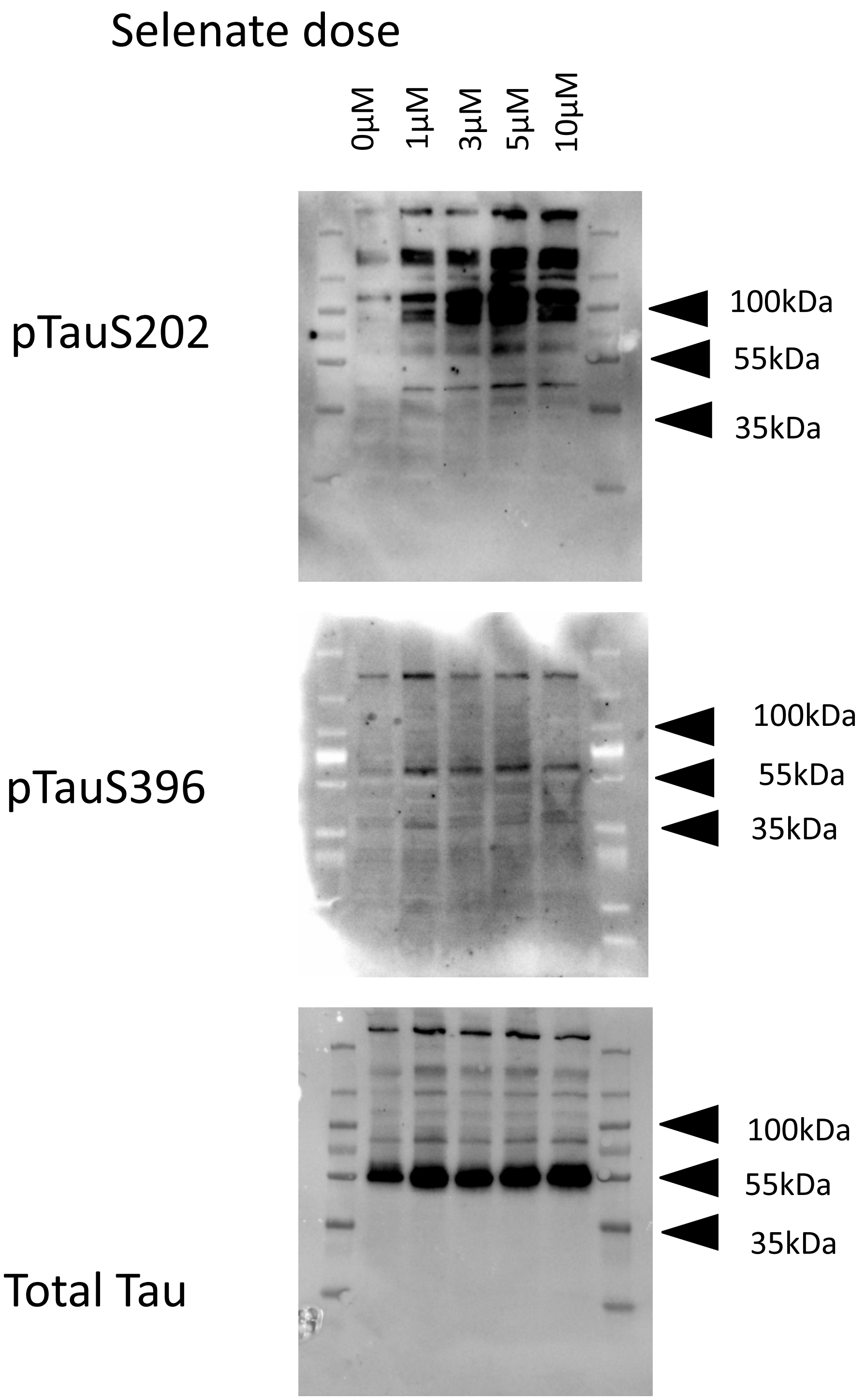

Supplement: Supplementary file 1 [file ijms-20-00844-s001.pdf]
